# Smoothened as a new therapeutic target for human osteosarcoma

**DOI:** 10.1186/1476-4598-9-5

**Published:** 2010-01-12

**Authors:** Masataka Hirotsu, Takao Setoguchi, Hiromi Sasaki, Yukihiro Matsunoshita, Hui Gao, Hiroko Nagao, Osamu Kunigou, Setsuro Komiya

**Affiliations:** 1 Department of Orthopaedic Surgery, Graduate School of Medical and Dental Sciences, Kagoshima University, Kagoshima, 890-8520, Japan

## Abstract

**Background:**

The Hedgehog signaling pathway functions as an organizer in embryonic development. Recent studies have demonstrated constitutive activation of Hedgehog pathway in various types of malignancies. However, it remains unclear how Hedgehog pathway is involved in the pathogenesis of osteosarcoma. To explore the involvement of aberrant Hedgehog pathway in the pathogenesis of osteosarcoma, we investigated the expression and activation of Hedgehog pathway in osteosarcoma and examined the effect of SMOOTHENED (SMO) inhibition.

**Results:**

To evaluate the expression of genes of Hedgehog pathway, we performed real-time PCR and immunohistochemistry using osteosarcoma cell lines and osteosarcoma biopsy specimens. To evaluate the effect of SMO inhibition, we did cell viability, colony formation, cell cycle *in vitro *and xenograft model *in vivo*. Real-time PCR revealed that osteosarcoma cell lines over-expressed *Sonic hedgehog*, *Indian hedgehog*, *PTCH1*, *SMO*, and *GLI*. Real-time PCR revealed over-expression of *SMO, PTCH1*, and *GLI2 *in osteosarcoma biopsy specimens. These findings showed that Hedgehog pathway is activated in osteosarcomas. Inhibition of SMO by cyclopamine, a specific inhibitor of SMO, slowed the growth of osteosarcoma in vitro. Cell cycle analysis revealed that cyclopamine promoted G1 arrest. Cyclopamine reduced the expression of accelerators of the cell cycle including cyclin D1, cyclin E1, SKP2, and pRb. On the other hand, p21^cip1 ^wprotein was up-regulated by cyclopamine treatment. In addition, knockdown of *SMO *by *SMO *shRNA prevents osteosarcoma growth in vitro and in vivo.

**Conclusions:**

These findings suggest that inactivation of SMO may be a useful approach to the treatment of patients with osteosarcoma.

## Background

Osteosarcoma is the most common primary bone malignant tumor occurring mainly in children [1]. After initial diagnosis is made by biopsy, treatment consists of preoperative chemotherapy, followed by definitive surgery and postoperative chemotherapy. Survival has improved over the past several decades. Indeed, patients with non-metastatic disease have a 70% chance of long-term survival. Unfortunately, patients with metastatic disease at diagnosis and those who have recurrent disease have a poor prognosis, with only 20% surviving at 5 years, indicating that new therapeutic options for them need to be actively explored. In cancer cells, dysregulation of cell division and apoptotic processes contribute to both drug resistance and metastatic potential [2,3]. It has been reported that inactivation of the cell cycle regulatory pathway centered around the Rb gene is a critical step in the pathogenesis of osteosarcoma [4]. Although such dysregulation may constitute a potent source of new therapeutic targets, the molecular mechanisms of regulation of osteosarcoma cell proliferation are largely unknown.

Hedgehog (Hh) pathway has been implicated in different aspects of animal development, acting through several components, including the transmembrane proteins PATCHED (PTCH1) and SMOOTHENED (SMO), to activate the GLI zinc-finger transcription factors [5,6]. Hh pathway is critical for many processes during embryonic and postnatal development, including proliferation, differentiation, specification of cell fate, left-right asymmetry, and morphogenesis [7]. Sporadic and familial mutations in the Hh pathway genes, PTCH1, suppressor-of-fused, and SMO, leading to elevated expression of downstream target genes including GLI, have been reported in basal cell carcinoma and the pediatric brain tumor medulloblastoma [8,9]. In addition, the growth of many cancers has been suggested to depend on continuous Hh pathway even in the absence of activating mutations in the pathway (reviewed in ref. [10]).

To explore the involvement of Hh pathway in the pathogenesis of osteosarcoma, we investigated the expression and activation of the Hh pathway genes in osteosarcoma and examined the effect of inhibition of SMO by cyclopamine, a specific inhibitor of SMO [11] or *SMO *shRNA.

## Results

### Over-expression of Hh-GLI pathway molecules in osteosarcoma

**Figure 1:**
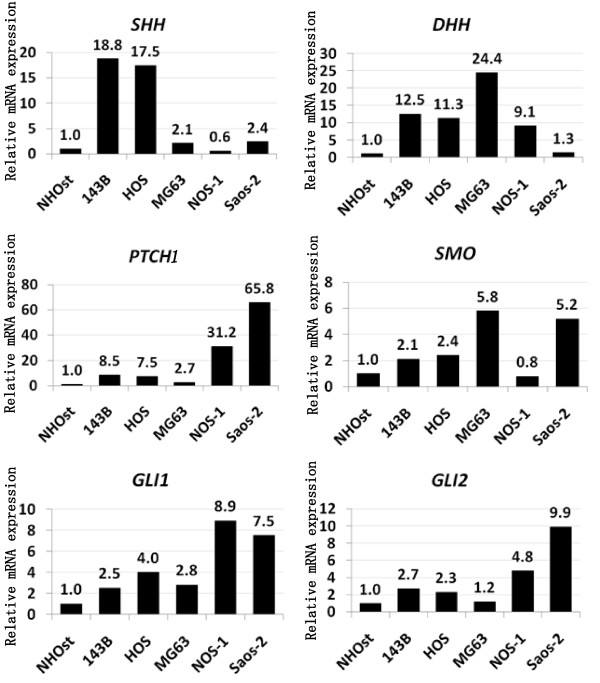
**Expression of activated Hh-GLI pathway molecules**. Total RNA extracted from osteosarcoma cell lines were used for real-time PCR. Real-time PCR revealed that 4 of 5 human osteosarcoma cell lines increased *Sonic Hedgehog *(*SHH*) 2.1- to 18.8-fold (Fig. 1). In addition, 5 of 5 osteosarcoma cell lines increased *Desert Hedgehog *1.3- to 24.4-fold (Fig. 1). To further examine Hh pathway molecules expression, we performed real-time PCR for Hh receptors and Hh target genes. *PTCH1 *was up-regulated 2.7-to 65.8-fold in 5 of 5 human osteosarcoma cell lines. *SMO *was up-regulated 2.1-to 5.8-fold in 4 of 5 human osteosarcoma cell lines. *SMO *was up-regulated 2.1-to 5.8-fold in 4 of 5 human osteosarcoma cell lines. *GLI1 *was up-regulated 2.5-to 8.9-fold in 5 of 5 human osteosarcoma cell lines. *GLI2 *was up-regulated 1.2-to 9.9-fold in 5 of 5 human osteosarcoma cell lines. The comparative Ct (ΔΔCt) method was used to determine fold change in expression using *βII-microglobulin*, *GAPDH *or *ACTB*. Each sample was run minimally at three concentrations in triplicate (error bar means S.D.). The experiment was triplicate with similar results.

**Figure 2:**
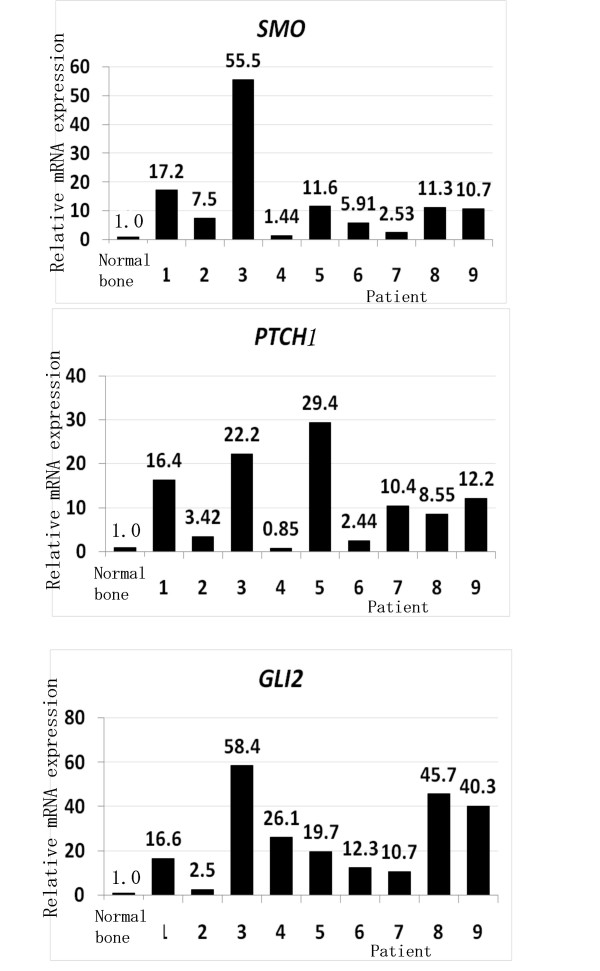
**Activation of Hh pathway in patient' biopsy samples**. Real-time PCR revealed that 9 of 9 human biopsy specimens of osteosarcoma increased *SMO *1.44- to 55.5-fold. Real-time PCR revealed that expression of *PTCH1 *was increased in 8 of 9 patients' biopsy samples 2.44- to 29.4-fold. *GLI2 *was up-regulated 2.5-to 58.4-fold in 9 of 9 human biopsy specimens of osteosarcoma. The comparative Ct (ΔΔCt) method was used to determine fold change in expression using *βII-microglobulin*, *ACTB*, and *GAPDH*. Each sample was run minimally at three concentrations in triplicate (error bar means S.D.). The experiment was triplicate with similar results.

To examine the role of Hhï¿½GLI pathway in osteosarcoma, we tested for the expression of Hh in osteosarcoma cell lines. Real-time PCR revealed that 4 of 5 human osteosarcoma cell lines increased *Sonic Hedgehog *(*SHH*) 2.1- to 18.8-fold (Fig. 1). In addition, 5 of 5 osteosarcoma cell lines increased *Desert Hedgehog *1.3- to 24.4-fold (Fig. 1). To further examine Hh pathway molecules expression, we performed real-time PCR for Hh receptors and Hh target genes. *PTCH1 *was up-regulated 2.7-to 65.8-fold in 5 of 5 human osteosarcoma cell lines. *SMO *was up-regulated 2.1-to 5.8-fold in 4 of 5 human osteosarcoma cell lines. *SMO *was up-regulated 2.1-to 5.8-fold in 4 of 5 human osteosarcoma cell lines. *GLI1 *was up-regulated 2.5-to 8.9-fold in 5 of 5 human osteosarcoma cell lines. *GLI2 *was up-regulated 1.2-to 9.9-fold in 5 of 5 human osteosarcoma cell lines. To extend these findings, we performed immunocytochemistry for SMO and GLI2, and found that only osteosarcoma cells expressed detectable levels of SMO and GLI2. GLI2 was located in the nuclei of osteosarcoma cells (see additional file 1). We next examined *SMO *expression in osteosarcoma patient' biopsy specimens. Real-time PCR revealed that 9 of 9 human biopsy specimens of osteosarcoma increased *SMO *1.44- to 55.5-fold (Fig. 2). In addition, real-time PCR revealed that expression of *PTCH1 *was increased in 8 of 9 patients' biopsy samples 2.44- to 29.4-fold (Fig. 2). *GLI2 *was up-regulated 2.5-to 58.4-fold in 9 of 9 human biopsy specimens of osteosarcoma (Fig. 2). Of most importance was the finding that markers of active Hhï¿½GLI signaling, *GLI2 *and *PTCH1 *were consistently up-regulated in the examined osteosarcoma cells, demonstrating the aberrant Hh-GLI pathway activation [12-14]. Our findings suggest that Hh-GLI signaling is active in osteosarcomas.

### Inhibition of SMO prevents osteosarcoma growth in vitro

**Figure 3:**
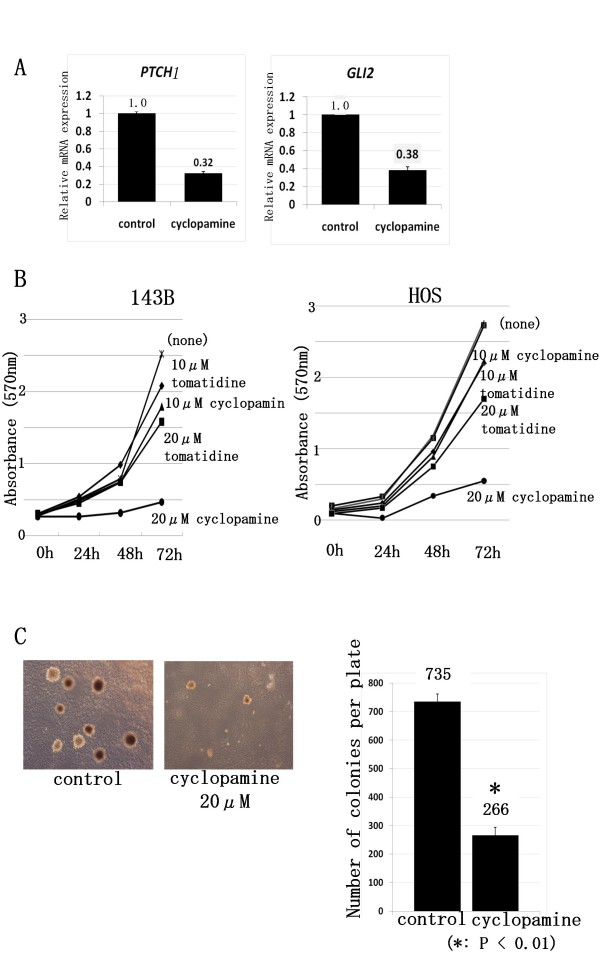
**Inhibition of Hh pathway prevents osteosarcoma growth in vitro**. A, We performed real-time PCR to determine which concentration of cyclopamine effectively inhibited Hh-GLI activity in osteosarcoma cells, and then measured the expression of the Hh-GLI pathway target *PTCH1 *and *GLI2*. Cyclopamine at 20 μM reduced mRNA levels of PTCH1 in 143B cell (error bar means S.D.). The comparative Ct (ΔΔCt) method was used to determine fold change in expression using *ACTB*. Each sample was run minimally at three concentrations in triplicate (error bar means S.D.). The experiment was triplicate with similar results. B, Growth of viable 143B and HOS cells over 3 days was slowed in dose-dependent fashion by cyclopamine treatment. The experiment was triplicate with similar results. C, Colony formation assay revealed cyclopamine reduced colony formation in soft agar. The experiment was triplicate with similar results. (*: P < 0.01) (error bar means S.D.)

To determine whether activation of Hh-GLI signaling is required for osteosarcoma cell growth, we used cyclopamine, a pharmacological agent known to effectively block Hh-GLI signaling by inhibiting SMO activation [11]. We performed real-time PCR to determine whether cyclopamine effectively inhibited the expression of the GLI target gene *PTCH1 *and *GLI2 *[14]. Cyclopamine at 20 μM reduced mRNA levels of *PTCH1 *and *GLI2 *in osteosarcoma cells by more than 60%, consistent with the expected down-regulation of Hh-GLI signaling (Fig. 3A). As cyclopamine was used to prevent cancer cells growth at 10 to 20 μM [15-17] we decided 20 μM was appropriate concentration for osteosarcoma. MTT assay showed that cyclopamine slowed the growth of HOS and 143B in dose-dependent fashion (Fig. 3B). On the other hand, MTT assay showed that proliferation of osteosarcoma cells was enhanced by SHH. We next used a clonogenic assay to determine whether cells capable of forming anchorage-independent colonies were depleted by cyclopamine. This assay revealed cyclopamine reduced colony formation in soft agar (Fig. 3C). These findings suggest that inhibition of SMO inhibited osteosarcoma growth in vitro.

### Hh signaling regulates cell cycle of osteosarcoma

**Figure 4:**
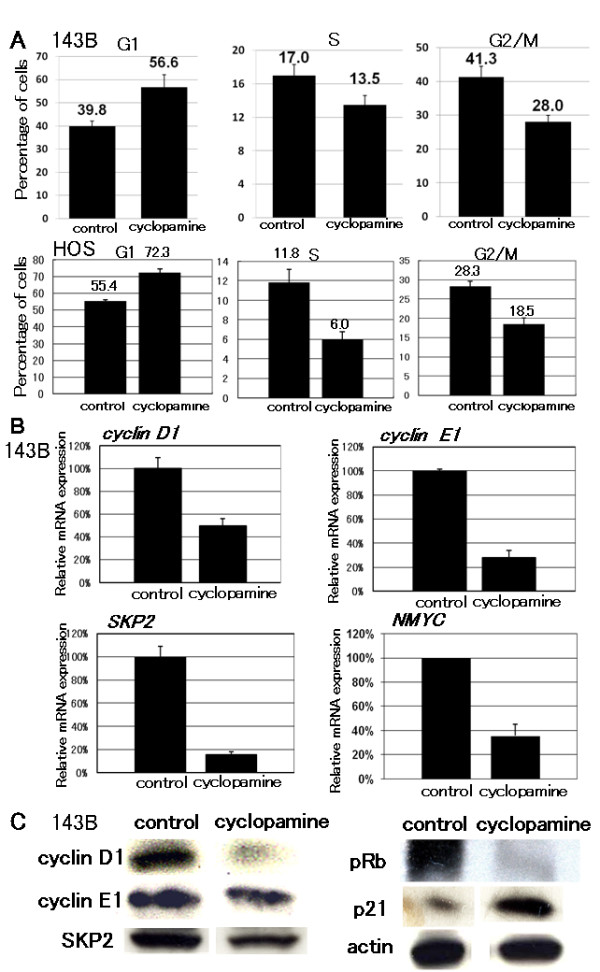
**Cyclopamine treatment promotes G1 arrest**. A, HOS and143B cells were treated with 10 μM cyclopamine. After 48-hour treatment cells were collected and subjected to cell cycle analysis. When 143B cells were cultured without cyclopamine, 39.8% of cells were in G1 phase. On the other hand, when cultured with cyclopamine, 56.6% of cells were in G1 phase. In the case of HOS cells cultured without GSI, 55.4% of cells were in G1 phase, while 72.3% of cells were in G1 phase when treated with cyclopamine (error bar means S.D.). B, Real-time PCR was performed to quantify mRNAs of cell cycle related genes. Twenty-four-hour treatment with cyclopamine reduced levels of *cyclin D1*, *Cyclin E1*, *SKP2*, and *NMYC *transcription (error bar means S.D.). The comparative Ct (ΔΔCt) method was used to determine fold change in expression using *βII-microglobulin *and *GAPDH*. Each sample was run minimally at three concentrations in triplicate (error bar means S.D.). The experiment was triplicate with similar results. C, Western blot analysis of levels of cell cycle-related genes. Forty-eight-hour treatment with cyclopamine reduced levels of expression of cyclin D1, cyclin E1, SKP2, and phosphprylated RB (pRb) proteins. Expression of P21^cip1 ^protein was upregulated by cyclopamine treatment. The experiment was triplicate with similar results (cyclopamine: 10 μM).

We examined cell cycle characteristics by flow cytometry. Of 143B cells cultured without cyclopamine, 39.8% of cells were in G1 phase, while 56.6% of cells were in G1 phase following treatment with cyclopamine. In the case of HOS cells were cultured without cyclopamine, 55.4% cells were in G1 phase. On the other hand, when cultured with cyclopamine, 72.3% of cells were in G1 phase (Fig. 4A). These findings suggested that cyclopamine promoted G1 arrest. We then examined the transcription of cell cycle-related genes. Real-time PCR revealed that cyclopamine prevented the transcription of accelerators of the cell cycle including *cyclin D1*, *cyclin E1*, *SKP2*, and *NMYC *(Fig. 4B). In mammalian cells, cyclin D, cyclin E, and p21^cip1 ^are short-lived proteins that are controlled by ubiquitin-dependent proteolysis. We performed western blot analysis to determine protein levels, and found that cyclopamine reduced the levels of expression of cyclin D1 and cyclin E1 proteins. Cyclopamine also reduced the levels of expression of cyclin D1, cyclin E1, pRb, and SKP2 proteins (Fig. 4C). We next examined the expression of p21^cip1^, and found that p21^cip1 ^protein was up-regulated by cyclopamine treatment (Fig. 4C). These findings suggested that cyclopamine promoted G1 arrest by inhibition of G1-S phase progression. These findings suggest that inhibition of SMO inhibited osteosarcoma growth via cell cycle regulation.

### Knock down of SMO prevents osteosarcoma growth in vivo

**Figure 5:**
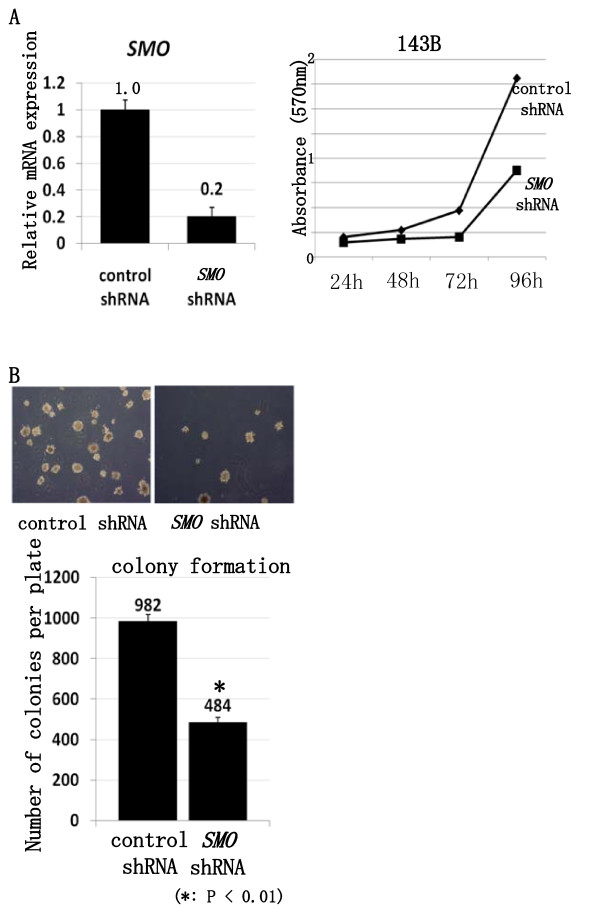
**Knock down of *SMO *by *SMO *shRNA prevents osteosarcoma growth in vitro**. A, Real-time PCR revealed that *SMO *shRNA effectively knock down *SMO *mRNA. (error bar means S.D.). The comparative Ct (ΔΔCt) method was used to determine fold change in expression using ACTB. Each sample was run minimally at three concentrations in triplicate (error bar means S.D.). The experiment was triplicate with similar results. B, Growth of viable 143B cells over 4 days was slowed by *SMO *shRNA. The experiment was triplicate with similar results. C, Colony formation assay revealed that *SMO *shRNA reduced colony formation in soft agar. The experiment was triplicate with similar results. (*: P < 0.01) (Error bar means S.D.)

**Figure 6:**
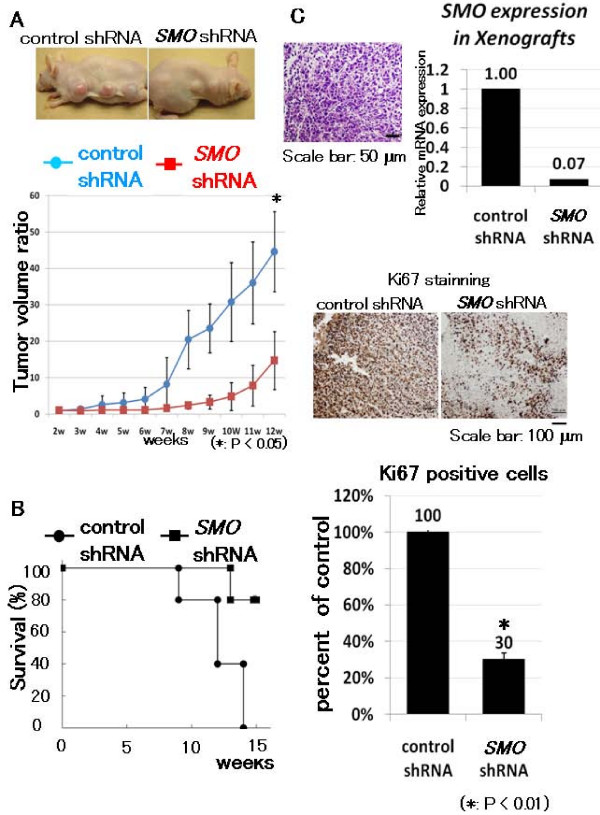
***SMO *shRNA prevents osteosarcoma xenograft growth in vivo and prolongs survival**. A, *SMO *shRNA or control shRNA transfected 143B cells (1 × 10^6^) were inoculated subcutaneously. Established 143B tumors were measured. The tumor volume at day 7 was set at 1, and tumor volumes at subsequent time points were calculated. *SMO *shRNA inhibited tumor growth at 8 weeks compared to control shRNA. B, Kaplan-Meier survival curves from *SMO *shRNA groups and control shRNA. Kaplan-Meier analysis showed that *SMO *shRNA conferred a significant survival benefit (n = 6, p < 0.05). C, Immunohistochemical examination of ki67 was performed in xenograft tumors. *SMO *shRNA decreased *SMO *RNA in vivo. Ki67 staining revealed that proliferation of osteosarcoma cells was decreased by GSI treatment. The number of Ki67-positive cells was decreased to 30% of control revel by *SMO *shRNA (error bar means S.D.) (*: P < 0.01).

To confirm the effect of SMO suppression, we examined the effect of *SMO *shRNA. 143B was transfected with control shRNA or *SMO *shRNA. *SMO *shRNA reduced the expression of *SMO *mRNA (Fig. 5A). MTT assay revealed that knock-down of *SMO *prevented osteosarcoma growth in vitro (Fig. 5A). We next used a clonogenic assay to determine whether cells capable of forming anchorage-independent colonies were depleted by *SMO *shRNA. This assay revealed *SMO *shRNA reduced colony formation in soft agar (Fig. 5B). These findings show that suppression of SMO prevents osteosarcoma growth in vitro. We then examined the transcription of cell cycle-related genes. Real-time PCR revealed that *SMO *shRNA prevented the transcription of accelerators of the cell cycle including *cyclin D1*, *cyclin E1*, *SKP2*, and *E2F1 *(see additional file 2). To examine the in vivo effect of *SMO *shRNA, nude mice were inoculated with control shRNA or *SMO *shRNA transfected 143B osteosarcoma cells intradermally. Results demonstrated significant inhibition of tumor growth *SMO *shRNA versus control shRNA (Fig. 6A, B). Kaplan-Meier analysis showed that *SMO *shRNA conferred a significant survival benefit (Fig. 6B). Next, we performed real-time PCR using formed tumors. Real-time PCR revealed that transcription of *GLI1*, *GLI2*, and PTCH1 was decreased in tumors formed by *SMO *shRNA-transfected 143B. These findings showed that *SMO *shRNA prevented the transcription of Hh target genes in vivo. In addition, *SMO *shRNA prevented the transcription of accelerators of the cell cycle including *cyclin E1*, *SKP2*, and *E2F1 *(see additional file 3). Histological analysis indicated that *SMO *shRNA prevented cell proliferation. The control tumors exhibited a number of cells positive for Ki67, a marker of cell proliferation. In contrast, *SMO *shRNA transfected tumors exhibited little evidence of proliferation, as evidenced by lack of Ki67 positivity. The number of Ki67-positive cells was decreased to 30% of control revel by *SMO *shRNA (Fig. 6C). These findings suggest that inhibition of SMO prevents osteosarcoma growth by cell cycle regulation in vivo.

## Discussion

Although the role of Hh signaling in various cancers [18-21], it's role in the pathogenesis of osteosarcoma has not been reported. In the present study, we found that *Shh*, *Dhh*, *PTCH1*, *SMO, GLI1 *and *GLI2 *transcripts were over-expressed in osteosarcoma cell line. In addition, *SMO, PTCH1*, and *GLI2 *were over-expressed in osteosarcoma biopsy specimens'. In general, it is accepted that enhanced Hh pathway activation leads to downstream expression of target genes including *PTCH1 *and *GLI*, and hence, the levels of these transcripts are often used as surrogate markers of Hh pathway activity [22]. In addition, SHH promoted osteosarcoma cells proliferation. Our findings suggest that Hh pathway is activated in osteosarcomas. On the other hand, *GLI1 *was down-regulated in human osteosarcoma biopsy specimens (data not shown). The reason for *GLI1 *down-regulation could not be determined. One possibility is that the *GLI1 *promoter is inactivated in human osteosarcomas by epigenetic modification. We found that GLI1 promoter contains a CG-rich region. Wong et al. reported that Hh pathway activity down-stream of SMO is mediated by GLI2 [23]. These data suggest that Hh activity down-stream of SMO is mediated by GLI2 instead of GLI1 in osteosarcoma.

SMO is a central transducer of the Hh signal and important anticancer drug target [11,14,19,22,24-33]. Warzecha et al reported that cyclopamine is able to inhibit proliferation of osteosarcoma cell lines [34]. In agreement with their findings, our results showed that inhibition of SMO by cyclopamine or *SMO *shRNA is efficient in suppressing tumourigenic properties of osteosarcoma cells both in vitro and in vivo. We used cyclopamine to inhibit SMO in xenograft model at first. We performed that treatment with 25 mg/kg cyclopamine reduced numbers of ki67-positive cells (see additional file 4). These findings suggest that inhibition of SMO prevents osteosarcoma growth by cell cycle regulation in vivo. Although it appeared that osteosarcoma growth was prevented by cyclopamine, all mice died for undetermined reasons by 1 month after cyclopamine treatment (data not shown). We next performed 10 mg/kg cyclopamine treatment, and found no difference in osteosarcoma growth between cyclopamine treatment and the control group (data not shown). Unfortunately, a therapeutic dose of this agent in the 143B xenograft model could not be obtained. It has been reported that cyclopamine might not be a good candidate for a drug in the treatment of malignant tumors because it had several serious side effects in young mice, including weight loss and dehydration, suggesting that it may not be possible to achieve a therapeutic dose in our xenograft model system [28,35]. In efforts to solve these problems, we used *SMO *shRNA. *SMO *shRNA inhibited osteosarcoma growth. Kaplan-Meier analysis showed that *SMO *shRNA conferred a significant survival benefit. It was reported that administration of RNAi resulted in silencing of the target genes in vivo [36-41]. These findings demonstrate the therapeutic potential of *SMO *shRNA for the treatment of osteosarcoma. Although SMO is the major signal transducer of the Hh pathway, SMO inhibition suppresses tumorigenesis by down-regulation of β-catenin mediated Wnt signaling [42]. It was reported that deregulation of β-catenin signaling is implicated in the pathogenesis of osteosarcoma [43,44]. Further examination might be needed the relationship between SMO inhibition and Wnt-β-catenin signaling in osteosarcoma.

Cyclopamine promoted G1 arrest in osteosarcoma in vitro. We also found that cyclopamine treatment regulated the expression of cell cycle regulators. Quantitative real-time PCR and western blot analysis revealed that cyclin D1, E1, SKP2, and pRB were down-regulated upon SMO inhibition with cyclopamine. Cyclin D1, cyclin E1, SKP2, and pRb have been reported to promote G1-S phase progression [45-48]. Our findings suggest that cyclopamine promoted cell cycle arrest via down-regulation of cyclins and pRb. It has been reported that cyclin D1 and cyclin E1 are direct targets of Hh signaling [49,50]. GLI2 mediated the mitogenic effects of Shh by transcriptional activation of cyclin D1 and cyclin D2 in developing hair follicles [51]. Our findings are consistent with the results of these previous studies. We showed that cyclopamine decreased the transcription of *SKP2*. The relationship between Hh signaling and SKP2 have not been reported. We attempted to find a GLI binding site (GACCACCCA) in the -1000 to +20 region of the 5' flanking sequence of *SKP2*, but found no GLI binding consensus sequence. These findings suggest that transcription of *SKP2 *might not be regulated by GLI. It has been reported that the *SKP2 *gene contains a functional E2F response element and is transcribed by E2F1 [52]. *E2F1 *is an early transcriptional target of GLI2 [53]. In addition, *E2F1 *transcription is activated by Rb phosphorylation. Our findings suggest that down-regulation of E2F1 and pRb indirectly reduced the transcription of *SKP2*. In addition, we showed that cyclopamine treatment promoted p21^Cip1 ^up-regulation. p21^cip1 ^can bind to various cyclin dependent kinases and that it inhibits their kinase activity. Our findings suggest that inhibition of the Hh pathway reduces the expression of the *SKP2 *subunit of the ubiquitin-ligase complex SCF^SKP2^, which in turn inhibit proteasomeï¿½mediated degradation of p21^Cip1 ^and promote cell cycle arrest.

It has been reported that cyclopamine treatment induced apoptosis in tumor cells [20,32,54]. We performed apoptosis assay, but could not detect apoptosis of 143B osteosarcoma cell line (data not shown). This finding may be the result of differences in cell viability between osteosarcoma and other cancer cell lines.

Several key signalling pathways, such as Hedgehog, Notch, Wnt and BMP-TGFbeta-Activin (bone morphogenetic protein-transforming growth factor-beta-Activin), are involved in most processes essential to the proper development of an embryo. It is also becoming increasingly clear that these pathways can have a crucial role in tumorigenesis (reviewed in [19]). We previously reported that activation of Notch signaling promote the progression of human osteosarcoma [55]. Additionally, some recent reports have provided evidence for direct interaction or cross-talk between these pathways (reviewed in [56]). Further examination should be performed to elucidate these pathways interaction in osteosarcoma pathogenesis.

Several recent papers have demonstrated that anti-tumor effect by SMO inhibitors are mostly due to their effect on stromal cells [57,58]. On the other hand some papers have reported that Hh signaling pathway is activated in cancer cells [14,17,21,23,59]. Although, there is a possibility that anti-osteosarcoma effect by cyclopamine was partially dependent to the effect on bone marrow stromal cell, anti-tumor effect of *SMO *shRNA revealed that inactivation of SMO directly inhibits osteosarcoma proliferation in vitro and in vivo.

The hypothesis that malignant tumours are generated by rare populations of Tumour-initiating cells (TIC), also called cancer stem cells, that are more tumourigenic than other cancer cells has gained increasing credence [31,60]. Some reports have shown the existence of TICs in bone and soft tissue sarcomas [61-65]. Magali et al. reported that loss of Smo causes depletion of TICs whereas constitutively active Smo augments TICs number and accelerates disease [20,66]. These data suggest that inhibition of Hh pathway might affect the proliferation of TICs of osteosarcoma.

In conclusion, our findings demonstrate that the Hh pathway is functionally activated in osteosarcoma. This novel finding improves understanding of osteosarcoma and may be important in understanding the proliferation of osteosarcoma cells. Our findings suggest that inactivation of SMO may be an attractive target for the treatment of patients with osteosarcoma.

## Methods

### Cell culture

HOS, 143B, MG63, and Saos-2 cells were purchased from the American Type Culture Collection (ATCC, Manassas, USA). NOS-1 was purchased from RIKEN cell bank (Tsukuba, Japan) [67]. Cells were grown in Dulbecco's modified Eagle's medium (DMEM) supplemented with 10% FBS, penicillin (100 U/ml), and streptomycin (100 μg/ml). Human osteoblast cells (NHOst) were purchased from Sanko Junyaku (Tokyo, Japan). Cells were cultured with OBM™ (Cambrex, East Rutherford, NJ, USA) or DMEM supplemented with 10% FBS. All cells were grown in a humidified atmosphere containing 5% CO_2 _at 37°C.

### Patient' specimens

All human osteosarcoma biopsy specimens were obtained from primary lesions. Biopsy was performed before chemotherapy or radio therapy for diagnostic purpose. Normal bone tissue was obtained from femur during total hip arthroplasty. The study protocol was approved by the institutional review board of the Kagoshima University. All patients and controls gave written informed consent.

### MTT assay

Cells were incubated with substrate for MTT (3-(4,5-dimethylthiazol-2-yl)-2,5-diphenyltetrazolium bromide) for 4 h, and washed with PBS and lysed to release formazan from cells. Then cells were analyzed in a Safire microplate reader (BIO-RAD, Hercules, CA, USA) at 562 nm. Cyclopamine and tomatidine was purchased from Funakoshi (Tokyo, Japan). 143B cell were serum starved for 12 h, and then cultured with recombinant human sonic hedgehog (R&D Systems, Minneapolis, Japan).

SMO shRNA was purchased from SABiosciences (Maryland, USA). *SMO *and control shRNAs were cloned into pGeneClip™ Neomycin Vector, which express shRNA under the control of the U1 promoter. Lipofection of shRNA was performed every other day as recommended in the supplier's protocol using FuGENE 6 (Roche, Basel, Switzerland).

### Colony formation assay

Colony formation assay was performed as previously described [68]. Briefly, cells were suspended in DMEM containing 0.33% agar and 10% fetal bovine serum and plated onto the bottom layer containing 0.5% agar. The cells were plated at a density of 5 × 10^3 ^per well in a 24-well plate, and colonies were counted 14 days later. Each condition was analyzed in triplicate, and all experiments were repeated three times.

### Real-time PCR

All primer sets amplified 100- to 200-bp fragments. Total RNA was extracted using the miR-Vana RNA isolation system or TRIzol (Invitrogen, Carlsbad, CA, USA). Reactions were run using SYBR Green (BIO-RAD, Hercules, CA, USA) on a MiniOpticon™ machine (BIO-RAD, Hercules, CA, USA). The comparative Ct (ΔΔCt) method was used to determine fold change in expression using *βII-microglobulin*, or *GAPDH*, or *ACTB*. Each sample was run at three concentrations in triplicate. The following primers were used. *Desert hedgehog*: 5-TGATGACCGAGCGTTGTAAG-3, 5-GCCAGCAACCCATACTTGTT-3; *Indian Hedgehog*: 5-ACTTCTGCCTGGTCCTGTTG-3, 5-AGCGATCTTGCCTTCATAGC-3; *Sonic hedgehog*: 5-ACCGAGGGCTGGGACGAAGA-3, 5-ATTTGGCCGCCACCGAGTT-3; *PATCHED*: 5-TAACGCTGCAACAACTCAGG-3, 5-GAAGGCTGTGACATTGCTGA-3; *SMOOTHENED*: 5-GGGAGGCTACTTCCTCATCC-3, 5-GGCAGCTGAAGGTAATGAGC-3; *GLI1*: 5-GTGCAAGTCAAGCCAGAACA-3, 5-ATAGGGGCCTGACTGGAGAT-3, *GLI2*: 5-CGACACCAGGAAGGAAGGTA-3, 5-AGAACGGAGGTAGTGCTCCA-3; *cyclin D1*: 5-ACAAACAGATCATCCGCAAACAC-3, 5-TGTTGGGGCTCCTCAGGTTC-3; *cyclin E1*: 5-CCACACCTGACAAAGAAGATGATGAC-3, 5-GAGCCTCTGGATGGTGCAATAAT-3; *SKP2*: 5-TGGGAATCTTTTCCTGTCTG-3, 5-GAACACTGAGACAGTATGCC-3; *NMYC*: 5-CTTCGGTCCAGCTTTCTCAC-3, 5-GTCCGAGCGTGTTCAATTTT-3; *βII-microgloblin*: 5-TCAATGTCGGATGGATGAAA-3, 5-GTGCTCGCGCTACTC TCTCT-3; *GAPDH*: 5-GAAGGTGAAGGTCGGAGTC-3, 5-GAAGATGGTGATGGGATTTC-3; *ACTB*: 5-AGAAAATCTGGCACCACACC-3, 5-AGAGGCGTACAGGGATAGCA-3.

### Immunohistochemistry

The following primary antibodies were used; anti-SMO (diluted 1:200, Santa Cruz, CA. U.S.A), anti-GLI2 (diluted 1:200, Abcam, Cambridge, UK), and anti-ki67 (Zymed laboratories, San Francisco, USA). The following secondary antibodies were used:; fluorescein-conjugated goat anti-mouse IgG antibody (diluted 1:200; Jackson ImmunoResearch, PA, USA) and rhodamine-conjugated donkey anti-rabbit IgG antibody (diluted 1:200; Chemicon, Temecula, CA). The cells were counterstained with Hoechst 33258 (Molecular Probes, Carlsbad, CA, USA) to identify nuclei. Immunohistochemistry with each second antibody alone without primary antibody was performed as a control.

### Western blot

Cells were lysed using NP40 lysis buffer (0.5% NP40, 10 mM Tris-HCl (pH 7.4), 150 mM NaCl, 3 mM pAPMSF (Wako Chemicals, Kanagawa, Japan), 5 mg/ml aprotinin (Sigma, StLouis, USA), 2 mM sodium orthovanadate (Wako Chemicals, Kanagawa, Japan), and 5 mM EDTA). Lysates were subjected to SDS-PAGE and subsequent immunoblotting with antibodies to cyclin D1, E1, p21, SKP2, and pRb (Santa Cruz, CA. U.S.A). Detection was performed using the ECL detection system (Amersham, Giles, UK).

### Animal experiments

143B cells (1 × 10^6^) were mixed with a collagen gel in a 1:1 volume, and were inoculated subcutaneously in 5-week-old nude mice. The mice were randomly assigned to receive either cyclopamine (25 mg/kg-10 mg/kg) or an equal volume of DMSO as control. Cyclopamine and saline solution were administered by intraperitoneal injection. The treatment with cyclopamine was initiated 1 week after tumor inoculation when the tumors had grown to visible size. The injections were repeated every other day. Tumor size was measured with calipers weekly, and tumor volume was calculated using a formula of LW^2^/2 (L and W representing the length and width of tumors). SMO shRNA (SABiosciences, Maryland, USA) transfected 143B cells (1 × 10^6^) or control shRNA (1 × 10^6^) cells were mixed with a collagen gel in a 1:1 volume, and were inoculated subcutaneously in 5-week-old nude mice. Tumor size was measured with calipers weekly, and tumor volume was calculated using a formula of LW^2^/2 (L and W representing the length and width of tumors). All experimental procedures were performed in compliance with the guiding principles for the Care and Use of Animals described in the American Journal of Physiology and with the Guidelines established by the Institute of Laboratory Animal Sciences, Faculty of Medicine, Kagoshima University. All efforts were made to minimize animal suffering, to reduce the number of animals used, and to utilize possible alternatives to in vivo techniques.

### Cell cycle analysis

Cell cycle analysis was performed by Reprocell (Tokyo, Japan). At 48 h after cyclopamine treatment, cells were collected by trypsinization and washed with DPBS. Cells were fixed in 70% (v/v) ethanol at 4°C, washed with PBS, and resuspended with 500 μl of staining solution [PBS pH 7.4, 100 μg/ml DNase-free RNase, 1 mg/ml propidium iodide]. Cells were then analyzed by flow cytometry using a FACS Vantage SE (Becton Dickinson, Franklin Lakes, NJ). Data were gated using pulse width and pulse area to exclude doublets, and the percentage of cells present in each phase of the cell cycle was calculated using FlowJo software (Tree Star, Ashland, OR, USA).

### Statistics and supplemental data

Each sample was analyzed in triplicate, and experiments were repeated three times. In all figures, error bars are standard divisions. All statistical analyses were performed using Microsoft Office Excel (Microsoft, Albuquerque, New Mexico, USA) and STASTISCA (StatSoft, Tulsa, OK, USA). Differences between mean values were evaluated by the unpaired *t*-test, and differences in frequencies by Fisher's exact test. Differences were considered significant at *P *< 0.05.

## List of abbreviations

(Hh): Hedgehog; (SMO): SMOOTHENED; (PTCH1): PATCHED; (SHH): Sonic hedgehog; (DHH): Desert hedgehog.

## Competing interests

The authors declare that they have no competing interests.

## Authors' contributions

TS was involved in the design and execution of the experiments, drafted the manuscript and contributed to the overall experimental design. MH conducted most of the experiments. HS was conducted a most of experiments. HG was conducted a part of experiments. YM was conducted a part of experiments. HN was conducted a part of experiments. OK was conducted a part of experiments. SK contributed to the overall experimental design. All authors read and approved the final manuscript.

## Supplementary Material

Additional file 1 **A, Immunohistochemical examination revealed that SMO was expressed on cytoplasm of 143B and GLI2 was localized in the nucleus of 143B.** B, MTT assay showed that Sonic hedgehog promote proliferation of osteosarcoma cells. The experiment was triplicate with similar results. Click here for file

Additional file 2 **Real-time PCR was performed to quantify mRNAs of cell cycle related genes.***SMO *shRNA reduced levels of *cyclin D1*, *cyclin E1*, *SKP2*, and *E2F1 *transcription (error bar means S.D.). The comparative Ct (ΔΔCt) method was used to determine fold change in expression using *ACTB*. The experiment was triplicate with similar results. Click here for file

Additional file 3 **We performed real-time PCR using formed tumors.** Real-time PCR revealed that transcription of *GLI1*, *GLI2*, and *PTCH1 *was decreased in tumors formed by *SMO *shRNA-transfected 143B. In addition, *SMO *shRNA reduced levels of *Cyclin E1*, *SKP2*, and *E2F1 *transcription. The comparative Ct (ΔΔCt) method was used to determine fold change in expression using *ACTB*. The experiment was triplicate with similar results. Click here for file

Additional file 4 **Cyclopamine prevents proliferation of osteosarcoma *in vivo*.** Immunohistochemical examination of ki67 was performed in xenograft tumors. Ki67 staining revealed that proliferation of osteosarcoma cells was decreased by cyclopamine treatment. The numbers of Ki67-positive cells was decreased to 50% of control revel by cyclopamine administration at day 14 (error bar means S.D.). Click here for file
