# Supplementary figures and images for: Smoothened as a new therapeutic target for human osteosarcoma
Source: Mol Cancer. 2010 Jan 12;9:5. doi: 10.1186/1476-4598-9-5 (PMC2818696; doi:10.1186/1476-4598-9-5)

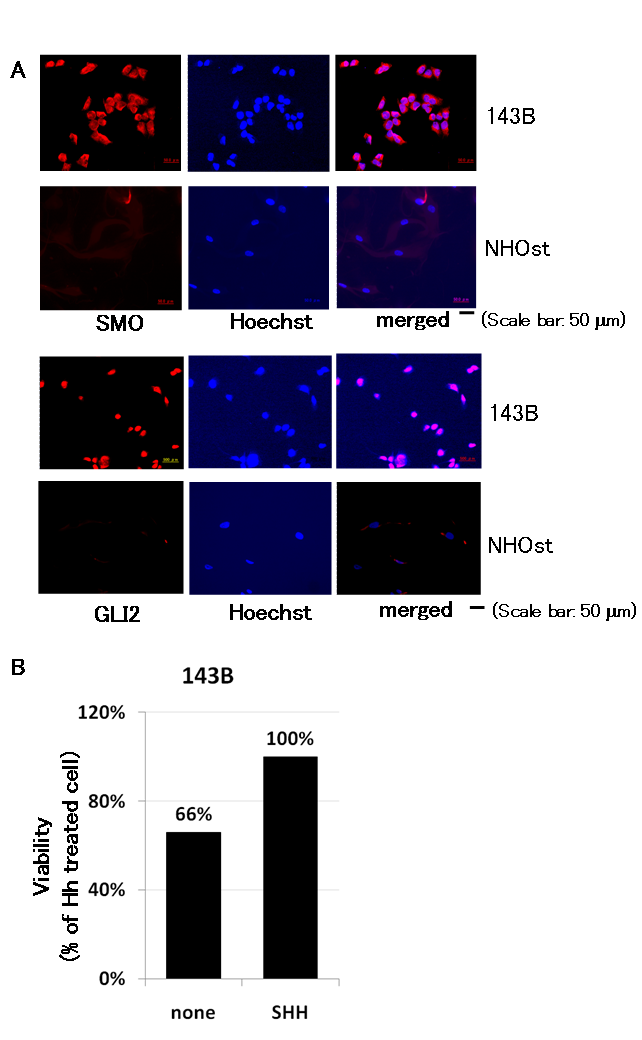

Supplement: Additional file 1 — A, Immunohistochemical examination revealed that SMO was expressed on cytoplasm of 143B and GLI2 was localized in the nucleus of 143B. B, MTT assay showed that Sonic hedgehog promote proliferation of osteosarcoma cells. The experiment was triplicate with similar results. [file 1476-4598-9-5-S1.TIFF]

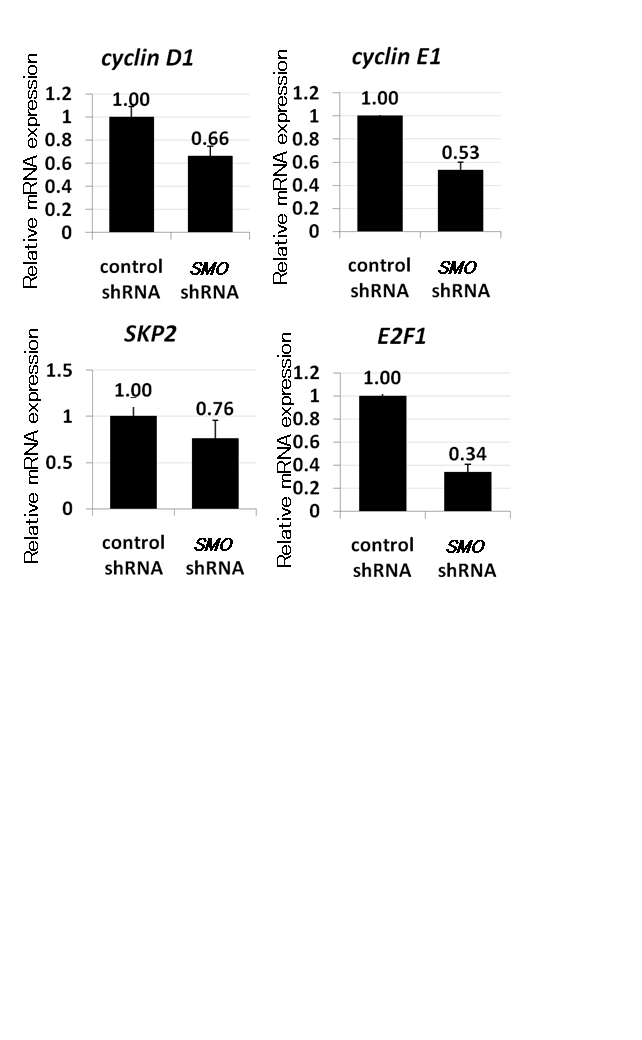

Supplement: Additional file 2 — Real-time PCR was performed to quantify mRNAs of cell cycle related genes.SMO shRNA reduced levels of cyclin D1, cyclin E1, SKP2, and E2F1 transcription (error bar means S.D.). The comparative Ct (ΔΔCt) method was used to determine fold change in expression using ACTB. The experiment was triplicate with similar results. [file 1476-4598-9-5-S2.TIFF]

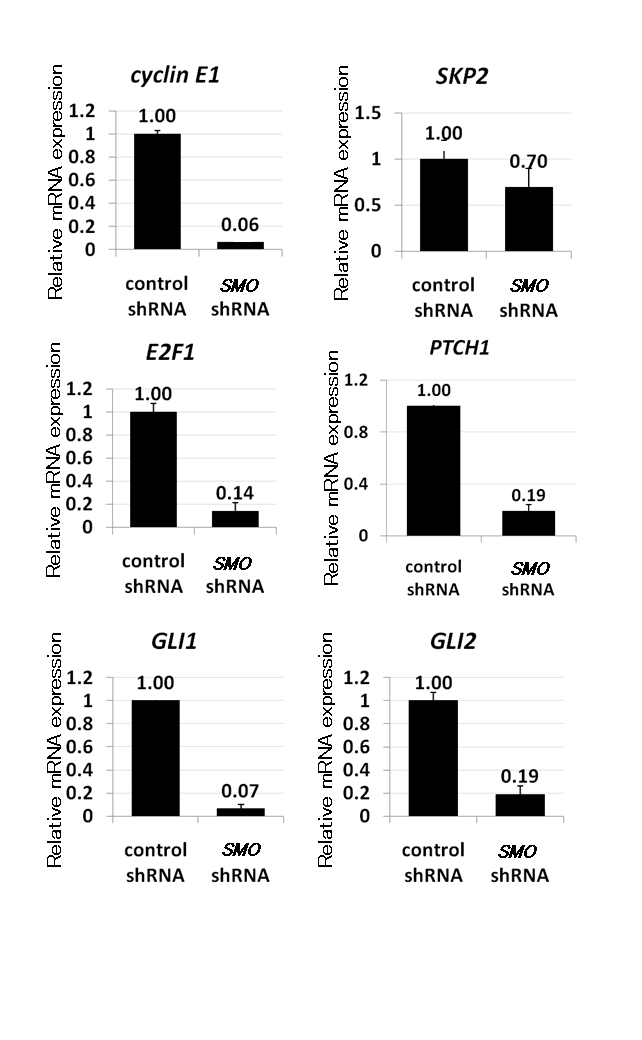

Supplement: Additional file 3 — We performed real-time PCR using formed tumors. Real-time PCR revealed that transcription of GLI1, GLI2, and PTCH1 was decreased in tumors formed by SMO shRNA-transfected 143B. In addition, SMO shRNA reduced levels of Cyclin E1, SKP2, and E2F1 transcription. The comparative Ct (ΔΔCt) method was used to determine fold change in expression using ACTB. The experiment was triplicate with similar results. [file 1476-4598-9-5-S3.TIFF]

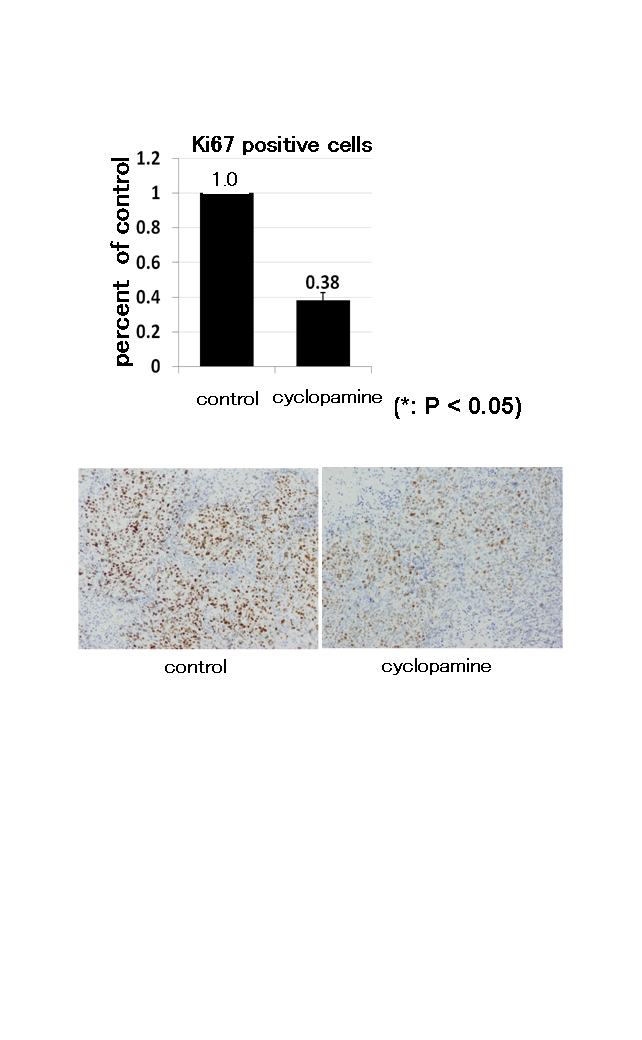

Supplement: Additional file 4 — Cyclopamine prevents proliferation of osteosarcoma in vivo. Immunohistochemical examination of ki67 was performed in xenograft tumors. Ki67 staining revealed that proliferation of osteosarcoma cells was decreased by cyclopamine treatment. The numbers of Ki67-positive cells was decreased to 50% of control revel by cyclopamine administration at day 14 (error bar means S.D.). [file 1476-4598-9-5-S4.TIFF]
